# Pancreatitis and atypical Kawasaki disease

**DOI:** 10.1186/1546-0096-8-8

**Published:** 2010-02-11

**Authors:** Dragan Prokic, Goran Ristic, Zoran Paunovic, Srdjan Pasic

**Affiliations:** 1 Department of Pediatric Gastroenterology, Mother and Child Health Institute, Medical School, University of Belgrade, Serbia; 2 Department of Immunology, Mother and Child Health Institute, Medical School, University of Belgrade, Serbia; 3 Department of Surgery, Mother and Child Health Institute, Medical School, University of Belgrade, Serbia

## Abstract

We report on pediatric patient with clinical and laboratory evidence of pancreatitis at onset of atypical Kawasaki disease (KD). In KD pancreatic inflammation was described previously, but clinical pancreatitis was rarely reported and its true incidence is unknown.

In febrile pediatric patients suspected to have KD, but not fulfilling classical diagnostic criteria, signs of pancreatic inflammation may help in making correct diagnosis. Further analysis of cases with atypical KD developing pancreatitis may reveal if signs of pancreatic inflammation can be used as supportive diagnostic finding.

## Case Report

A 6-year-old boy of Caucasian origin was referred to our Institute because of fever, rash and abdominal pain. He was seen in regional hospital with a 5-day history of fever, abdominal pain and vomiting and a 2-day history of scarlatiniform rash and oral hyperemia. He was treated with penicillin for suspected scarlet fever.

Physical examination at admission revealed abdominal tenderness to palpation, with the upper quadrants more tender than the lower. Abdominal X-ray showed distended bowel loops without obstruction. Abdominal ultrasound examination showed hydrops of the gallbladder and enlarged, edematous pancreas with small effusion in peritoneal cavity. Laboratory investigations at admission included: erythrocyte sedimentation rate 40 mm/hr, C-reactive protein 35 mg/l (normal < 10 mg/L), hemoglobin 112 g/l, WBC count 14.2 × 10^9^/l with 80% neutrophils; initial platelet count was 340 × 10^9^/l; total bilirubin, 65 μmol/l with conjugated bilirubin, 49 μmol/l; increased serum amylase of 168 IU/l (normal range, 40 - 110 IU/l) and urinary amylase,1317 IU/l (normal range 13-680 IU/l); lipase 95 IU/l (normal range, 8-57 IU/l), γ-glutamil transpeptidase 177 IU/l (normal range, 3- 22 IU/l), alanine aminotransferase, 84 IU/l (normal < 35 IU/l), aspartate aminotransferase, 167 IU/l (normal < 35 IU/l) and alkaline phosphatase, 437 IU/l were all elevated. Serologic tests to adenoviruses, enteroviruses, cytomegalovirus, Epstein-Barr virus or mumps were negative. Throat culture was negative and antistreptolysin titer was within normal values (less than 200 units). Routine urine examination revealed sterile pyuria. Heart ultrasound showed no abnormalities of coronary arteries.

On the seventh day of his disease intravenous immunoglobulin (IVIG) at a dose of 2 g/kg/body weight was given over the 48 hours and the patient became afebrile 48 hours later. In the second week of the disease thrombocytosis (platelet count, 600 × 10^9^/l) and periungval desquamation of his fingers and toes were observed. Repeated heart ultrasound showed no abnormalities of coronary arteries. Serum amylase and lipase values returned to normal eight days after admission.

## Discussion

Kawasaki disease (KD) is a systemic vasculitis of unknown etiology [1]. Atypical KD is defined as one in which atypical symptoms/signs not belonging to the classical criteria of KD herald the onset of the disease [2]. Children with atypical KD may present with acute abdominal symptoms, meningeal irritation, pneumonia or renal impairment [3,4]. Inflammatory lesions in pancreas have been previously described in KD but clinical pancreatitis has been rarely reported [5-8].

Gastrointestinal symptoms, particularly hydrops of gallbladder and hepatic dysfunction are common findings in children with KD [2,3]. Stoler *et al*. first reported two patients with pancreatitis during the second week of the disease and suggested that pancreatitis should be considered in patients with severe abdominal symptoms [6]. Similar to our patient, Lanting *et al*. reported pancreatitis heralding KD in 5-year-old patient and suggested that KD should be included in differential diagnosis of acute pancreatitis in childhood [7]. Also, Blum-Hoffmann *et al*. reported pancreatitis with small peritoneal effusion in a 3-year-old girl with KD [8].

In KD presenting with abdominal pain, vomiting or jaundice the final diagnosis of KD may be delayed [3]. Zulian *et al*. reported ten patients with acute surgical abdomen at the onset of KD, and in nine patients laparatomy was performed leading to final diagnosis of gallbladder hydrops with cholestasis, paralytic ileus, small intestinal obstruction or appendicular vasculitis [3]. In the same study, in spite of severe abdominal symptoms leading to laparatomy, not a single patient had an evidence of pancreatitis [3].

Patients with incomplete KD usually exhibit less than required number of criteria at onset and for several days thereafter [4]. Atypical features may or may not be present. Our patient developed only three of the six classical criteria for KD but additional findings such as sterile pyuria led to final diagnosis. The use of IVIG in our patient led to rapid resolution of fever and abdominal pain. Pancreatitis in KD is characterized with vasculitis of the medium-sized arteries and veins [5]. Beneficial effect of IVIG in KD complicated with pancreatitis was previously explained by resolution of vasculitis of pancreatic blood vessels [8,9].

Recently, Asano *et al*. reported 2-year-old patient with KD and pancreatitis [9]. The same authors also detected asymptomatic increase of serum amylase in 12 (8.6%) of consecutive 138 patients with KD [9]. Except of one case with pancreatitis, analysis of isoenzymes in another four patients in this study showed that an increased amylase is of salivary origin [9]. Therefore, an increased serum amylase level is not infrequent finding in KD but diagnosis of pancreatitis should be supported by clinical symptoms or imaging studies. Also, measurement of serum lipase level, being more specific than hyperamilasemia, may be used for diagnosis of pancreatitis.

Atypical or incomplete forms of KD imply the need for new diagnostic tool [3,4]. In cases of atypical KD physicians often use additional findings, which are not specific for disease but which do occur in substantial number of patients [10]. These signs include cardiovascular, gastrointestinal, articular, urinary or neurological symptoms, as well as, ultrasound findings such as hydrops of the gallbladder or evidence of myo/pericarditis. The real incidence of symptomatic affection of pancreas in KD is not known. Moreover, young infants and toddlers with KD may appear irritable for various reasons (e.g. meningeal irritation, polyarthritis) so that abdominal symptoms caused by pancreatic inflammation may be overlooked. Further evaluation of atypical/incomplete cases of KD may show if pancreatitis may be used as supportive diagnostic finding.

In febrile pediatric patients suspected to have KD, but not fulfilling classical diagnostic criteria, signs of pancreatic inflammation may be helpful in making correct diagnosis.

## Consent

Writen informed consent has been obtained from the parents of the patient reported in this Ms.

## List of Abbreviations

KD: Kawasaki disease; IVIG: intravenous immunoglobulin.

## Competing interests

We, the authors declare that we have no competing interests. This article is own work by the authors, and it is not supported by any financial or other organization.

## Authors' contributions

DP, GR, ZP - substantial contribution to conception and design. Each of them participated sufficiently in preparation of this Ms. SP - made the final analysis and critical revision of this Ms. All the authors give the final approval for Ms. entitled "Pancreatitis and atypical KD" to be published.
